# A study protocol for a randomised controlled trial assessing the effects of daily supplementation with defatted almond protein powder on fitness training adaptations and metabolic health in overweight and obese post-menopausal women

**DOI:** 10.1186/s12937-026-01330-w

**Published:** 2026-05-20

**Authors:** Vy Tran, Charles S Urwin, Michael Tieland, Sze-Yen Tan, Jackson J Fyfe, Simon Feros, Clinton Bruce, Shaun Mason, Rhiannon Snipe, Giselle Allsopp, D. Lee Hamilton, Zoya Huschtscha

**Affiliations:** Institute for Physical Activity and Nutrition, Faculty of Health, School of Exercise and Nutrition Sciences, Deakin University, Geelong, Victoria 3216 Australia

**Keywords:** Menopause, Sarcopenia, Plant-based protein supplementation, Almond protein, Lean mass, Dietary fibre, Cardiometabolic health, Oral glucose tolerance

## Abstract

**Background:**

Sarcopenia, characterised by declines in muscle mass and function, is expected to impact many postmenopausal women due to ageing and sedentary lifestyles. Hormonal changes during menopause exacerbate loss of lean mass, visceral fat gain, and risk of cardiometabolic disease. Resistance and high-intensity interval training (HIIT) are effective countermeasures, with adequate protein and fibre intake essential to support favourable adaptations. However, many women fall short of these protein and fibre dietary recommendations, particularly at breakfast. Defatted almond protein powder (APP), a by-product of almond oil production, is rich in protein and fibre and may enhance exercise-induced adaptations, though its effects remain untested.

**Methods:**

This protocol outlines a 12-week study comprising a 10-week supervised resistance and HIIT intervention, with baseline and post-intervention assessments conducted over one-week periods immediately before and after the intervention. The randomised, double-blind, placebo-controlled trial will investigate the effects of defatted APP supplementation in postmenopausal women. We aim to recruit 48 participants who will be randomly allocated to receive either APP or an energy-matched placebo (maltodextrin). Primary outcomes include changes in total and regional lean mass which will be assessed using dual-energy X-ray absorptiometry (DXA). Secondary outcomes include strength, assessed via 3-repetition maximum (3-RM) chest press and leg press, and maximal handgrip strength; cardiovascular fitness (VO₂peak); total body fat (absolute and %), blood lipids; blood glucose; insulin response; appetite; supplement acceptability; and dietary intake. Data will be analysed using linear regression and mixed-effects models under intention-to-treat and per-protocol frameworks.

**Discussion:**

This study will be the first to examine the effects of daily supplementation with APP on musculoskeletal and metabolic outcomes in postmenopausal women undergoing structured and supervised exercise training. Findings of this study may assist in developing applications for high protein/fibre by-product from the nut/seed oil industry and determine if supplementation with this almond derived product is an effective nutritional strategy to support training adaptations to strength and HIIT training in ageing women.

**Trial registration:**

ANZCTR: ACTRN12625000133437 (Date Registered: 06 February 2025). Registration URL: ANZCTR Trial Registration.

**Ethics: DUHREC: (2024/HE000126).**

**Version: 2.0 date (16/02/2026).**

**Supplementary Information:**

The online version contains supplementary material available at 10.1186/s12937-026-01330-w.

## Introduction

### Background and rationale

The global number of individuals aged ≥ 60 years is projected to double from 1.1 billion in 2023, to more than 2.1 billion by 2050 [1]. While females tend to live longer than males, they face nearly twice the risk of disability and incur a greater burden of chronic disease [2, 3]. This sex-based difference in disease burden may be driven (at least in part) by menopause. Menopause is associated with accelerated loss of fat-free mass and increases in visceral and subcutaneous adipose tissue [4–6]. These changes are linked to elevated cardiometabolic risk factors including increased blood pressure, low-density lipoprotein cholesterol (LDL-C), and obesity [5, 7]. Excess visceral fat contributes to cardiovascular disease through insulin resistance, systemic inflammation, and lipid dysregulation [8]. In parallel, ageing is associated with a progressive decline in strength, physical function and skeletal muscle mass (i.e., sarcopenia), which contributes to frailty, disability and loss of independence in older adults [9]. To mitigate these age- and menopause-related declines, evidence suggests that a combination of targeted exercise training and strategic nutrition approaches may support healthy aging [10, 11]. In particular, dietary protein and fibre intake may help preserve musculoskeletal and metabolic health respectively, in post-menopausal women [9, 12].

Exercise-based strategies, including resistance training and high-intensity interval training (HIIT) are effective in improving body composition, physical capacity and cardiometabolic health in postmenopausal women [13, 14]. Resistance training enhances muscular strength and lean mass in a dose-dependent manner, while also improving metabolic markers such as LDL-C [15, 16]. HIIT, characterised by alternating periods of vigorous intensity exercise and rest periods, improves cardiovascular fitness, and has been shown to reduce visceral fat and blood pressure, while also improving lipid profiles and blood glucose control in postmenopausal women [17, 18]. These adaptations may be enhanced by nutritional strategies that support muscle protein synthesis (MPS) and metabolic health following menopause.

While dietary protein is critical for maximising MPS in response to exercise, ageing is associated with anabolic resistance, a blunted sensitivity of skeletal muscle to protein intake [19–21]. To counteract anabolic resistance, daily protein intake recommendations for older adults have been revised upward to at least 1.2 g per kilogram (g/kg) of body mass (BM), with 20 to 30 g per meal suggested to maximise MPS, especially in the context of post-training recovery [22, 23]. A 2018 Australian national report estimated that the average protein intake for females aged 19 to 70 years was 79 g/day, equivalent to approximately 1.1 g/kgBM based on an average weight of 71 kg [19]. While this exceeds the Estimated Average Requirement (EAR) of 0.6–0.7 g/kgBM for nitrogen balance, it may still be insufficient to support muscle preservation and prevent sarcopenia in ageing populations [22]. Furthermore, protein intake is often skewed toward the evening meal, with breakfast and lunch typically providing only 10–18 g and 14–18 g, respectively, amounts that are suboptimal for stimulating MPS earlier in the day [19, 21, 24, 25]. Therefore, postmenopausal women may benefit from supplementing their diet with protein, particularly early in the day.

In addition to suboptimal protein intake, national survey data indicate that postmenopausal women are also consuming inadequate amounts of dietary fibre [21, 26]. Dietary fibre intakes for women > 50 years are reported as 21–22 g/day, which falls well below the suggested target of 28 g per day for optimal health benefits [27]. Soluble fibres slow gastric emptying and form viscous gels in the gut, reducing postprandial glucose and insulin spikes [28]. Fermentable fibres are metabolised by gut microbiota into short-chain fatty acids (SCFAs), such as butyrate, which have been linked to improved insulin sensitivity, reduced systemic inflammation, and better lipid metabolism [29, 30]. Fibre intake has also been associated with reduced LDL-C, enhanced satiety, and healthier body composition, all of which are important in reducing the risk of cardiovascular disease and type 2 diabetes risk in ageing women [31–33]. Therefore, optimising fibre intake may complement the benefits of exercise and protein intake in improving metabolic health and physical functional outcomes in postmenopausal women.

While animal-based proteins have traditionally been considered superior for MPS, plant-based proteins including soy, pea, and rice, can be equally effective if consumed in adequate amounts [34, 35]. Emerging evidence suggests that nut-based protein powders, such as defatted almond protein powder (APP), may provide an alternative plant protein source with additional cardiometabolic benefits [36–38]. APP is produced as a by-product of almond oil extraction, which results in a protein- and fibre-rich ingredient with a substantially reduced energy content [39]. This nutritional profile may be particularly beneficial for post-menopausal women, who typically have low energy requirements yet would benefit from additional protein. Despite the potential of APP as a nutritional supplement, its effects on exercise-induced adaptations remain unexplored.

## Objectives

The primary objective of this study is to evaluate the additive effects of defatted APP, beyond those induced by exercise training alone, on lean muscle mass in postmenopausal women undertaking a 10-week, fully supervised resistance and high-intensity interval training (HIIT) program.

Secondary outcome measures include between-group differences in muscular strength and aerobic fitness; body composition (including total and regional fat mass); cardiometabolic health markers, including blood glucose and insulin sensitivity, derived from an oral glucose tolerance test (OGTT) and calculated using validated surrogate indices (e.g. the Matsuda insulin sensitivity index and/or HOMA-IR derived from fasting values); lipid profiles; appetite regulation and taste preference; and dietary intake patterns, with a specific focus on protein and fibre intake.

Given that both groups complete an identical exercise intervention, all secondary outcomes will be interpreted as reflecting the incremental contribution of APP supplementation, relative to an energy-matched placebo, rather than the independent effects of exercise or fibre alone. Outcomes will be assessed at baseline and following the 10-week intervention.

### Methods: study design and setting

**Fig. 1:**
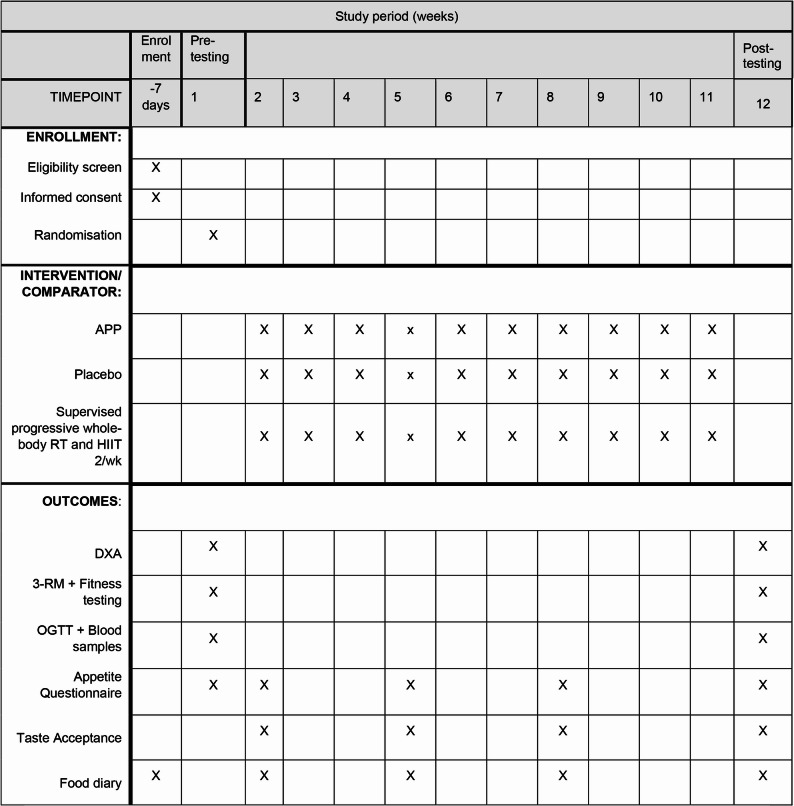
Schematic of experimental timeline illustrating the test weeks and the training weeks in addition to the test-day timeline illustrating the testing procedures. Abbreviations: 3-RM: 3 repetition maximum, APP: Almond Protein Powder, DXA: Dual-energy X-ray absorptiometry, HIIT: High-intensity interval training, OGTT: Oral glucose tolerance test, RT: resistance training. Figure adapted from [40]

This study is a 12-week, randomised, double-blind, placebo-controlled trial, which includes baseline and post-intervention assessment weeks (Week 1 and Week 12) and a 10-week supervised training and supplementation phase (Weeks 2–11) (Fig. 1). After the participants consume their first supplement in week 2, acceptance and supplement-related appetite responses will be measured.

### Overall experimental protocol

A detailed overview of the study timeline, including testing and training phases and procedures is presented in Fig. 1. The trial will take place at the Deakin University, Burwood campus (Melbourne, Australia), within a supervised, university-based laboratory setting and adheres to the Standard Protocol Items: Recommendations for Intervention Trials (SPIRIT, Appendix 1) [40]. Recruitment commenced in March 2025 and data collection is expected to finish in October 2026. This study design, including all participant consent forms and research tools, has been reviewed and approved by the Deakin University Human Research Ethics Committee (Approval Number: 2024/HE000126). Organisational consent was obtained to recruit within Deakin University. The trial has also been prospectively registered with the Australian New Zealand Clinical Trials Registry (ANZCTR) (ID: ACTRN12625000133437).

## Eligibility criteria

### Recruitment and consent

Participants will be recruited using physical posters displayed on university campuses, community centres, and fitness facilities, as well as targeted online advertisements via social media platforms and institutional mailing lists. Online advertisements will target women aged 50–65 years residing in metropolitan Melbourne and surrounding areas.

Interested individuals will be directed to an online expression of interest form via Research Electronic Data Capture (REDcap). This form includes a Plain Language Statement (PLS) outlining the purpose, procedures, risks, and benefits of the study. After reviewing the PLS, individuals will be required to provide electronic informed consent via REDcap before submitting any personal information or completing the eligibility screening questionnaire. Electronic consent will be obtained by participants actively selecting consent statements and providing an electronic signature, in accordance with Deakin University Human Research Ethics Committee (DUHREC) approved procedures. Eligible participants identified through the online screening process will be contacted by a member of the research team via email or phone to confirm eligibility, answer any additional questions, and schedule laboratory visits. At the first laboratory visit, participants will be given the opportunity to ask further questions, and verbal confirmation of consent will be reconfirmed prior to the commencement of any study procedures. Participants will be informed that participation is voluntary and that they may withdraw from the study at any time without penalty or consequence.

Participants will be eligible for inclusion into the study if they meet the following criteria:

- Aged 50–65 years old
- Classified as overweight (25–29.9 kg/m²) or obese class I (30–34.9 kg/m²)
- For participants who identify as being of Asian ethnicity, we will also apply WHO-recommended Asian-specific BMI thresholds to better reflect known differences in body composition and metabolic risk:
  - ◦ Overweight Asian-threshold: BMI 23.0–24.9 kg/m²
  - ◦ Obese Asian-threshold: BMI ≥ 25.0–29.9 kg/m² [41]
- Postmenopausal, defined as natural, medical or surgical menopause with no menstrual bleeding for ≥ 12 months.
- Not currently engaged in progressive resistance or balance/mobility training (≥ 1 session per week) for the past 6 months.
- Weight stable (no more than ≤ ± 2 kg weight change over the previous 2 months).
- Not currently using hormone therapy, or using hormone therapy continuously for ≥ 12 months.
- No history of nut allergies.
- No uncontrolled hypertension (defined as resting blood pressure ≥ 140/90 mmHg or requiring recent medication changes).
- No history of thyroid conditions, neuromuscular or skeletal disorders, or clotting disorders.
- Not undergoing treatment for conditions that affect muscle mass (e.g., cancer treatment, anabolic steroids).
- No chronic disease affecting nutrient absorption (e.g., cancer, diabetes, cardiovascular disease).
- No injury preventing exercise participation.
- Non-smokers.

Participants will complete a Physical Activity Readiness Questionnaire (PAR-Q) as a self-screening tool to identify individuals who may require medical clearance before engaging in physical activity. Individuals with known cardiovascular conditions, high-risk factors, or concerning responses in the PAR-Q may require clearance from a doctor before starting an exercise program.

To help promote study retention, participants will also receive a $100 electronic gift card upon the completion of the study. There is no patient or public involvement planned in the design, conduct, or reporting of this trial due to the controlled nature of the intervention and the need for standardised procedures.

## Methods

### Pre-testing standardisation procedures

Participants will attend the Deakin University, Burwood laboratory for two visits: pre-intervention (baseline) and post-intervention (12-weeks). All visits will be conducted following an overnight fast of at least 12 h.

To ensure adequate carbohydrate availability prior to the oral glucose tolerance test (OGTT) and to prevent a false-positive results [42], participants will be provided with a standardised pre-packaged evening meal to be consumed the night before each laboratory visit. Participants will be offered either a meat-based or vegetarian pre-packaged pasta dish, both matched for energy (1780–1900 kJ) and macronutrient composition, particularly carbohydrate content (~ 50–55 g), in accordance with best practice recommendations for OGTT preparation [42]. Participants will also be asked to refrain from drinking alcohol or exercising 24 h prior to each laboratory visit.

On the morning of testing, participants will be asked to consume 500 ml of plain water 45–60 min prior to their arrival. This hydration protocol is based on best practice guidelines to standardise hydration status for dual energy X-ray absorptiometry (DXA) scanning (DPX-IQ Lunar; Lunar Corporation, Madison, WI) [43]. In our previous studies, we have demonstrated that this approach results in consistent hydration status across testing sessions, with a coefficient of variation (CV) of 0.9% between pre- and post-intervention measures for lean muscle mass, measured using DXA, indicating excellent reproducibility [44].

Hydration status will be assessed using urine specific gravity (USG), measured via digital refractometry (PAL-10 S; Atago Co., Ltd., Tokyo, Japan). Following hydration assessment, the testing day will proceed in the following order: anthropometric measurements, DXA scan, fasting blood collection and OGTT, post-test standardised meal, followed by assessments of muscle strength and cardiorespiratory fitness. All efforts will be made to ensure the participants follow this order for both pre- and post-testing.

### Anthropometric assessment

At the commencement of the pre-testing visit, standardised anthropometric measurements including height (to the nearest 0.1 cm using a wall-mounted stadiometer) and body mass (to the nearest 0.1 kg using a calibrated digital scale (model UC-321; A&D Medical, Tokyo, Japan) will be recorded.

### Primary outcomes: body composition

The primary outcomes will be the mean percentage change (± standard deviation, SD) in total body and regional lean (arms and legs) and fat-free mass (FFM), assessed using DXA (Lunar Corp., Madison, WI). All DXA scans will be conducted by trained personnel experienced in the use of DXA technology and certified in accordance with standard operating procedures for body composition analysis. In addition to lean mass, total (kg) and relative (%) fat mass and visceral fat mass will also be reported as will the delta changes from pre-post training.

### Secondary outcomes

#### Blood collection and analysis

Following the DXA scan, participants will remain in a fasted state after which a cannula will be inserted in an antecubital vein for serial blood sampling. Cannulation and blood collection will be performed by a certified member of the research team (e.g., medical doctor or qualified personnel such as Huschtscha, Urwin, and Tran, all of whom are certified in venepuncture and cannulation procedures). A standard OGTT will then be administered, involving the ingestion of a 75 g/300 ml glucose solution (GTT Drink; Point of Care Diagnostics, Australia). Blood samples will be collected at 15-minute intervals from − 15 min to + 120 min post-ingestion to assess serum glucose and insulin concentrations over time.

In addition, a separate set of baseline fasting blood samples will be collected at -15 min for the analysis of blood lipid profiles, including insulin, high-density lipoprotein cholesterol (HDL-C), low-density lipoprotein cholesterol (LDL-C), triglycerides (TG), and total cholesterol (TC). Blood glucose will be analysed in-house using a HemoCue^®^ Glucose 201+ (Ängelholm, Sweden) in duplicate and the mean value will be reported. Serum insulin and other metabolic markers will be analysed by a certified commercial pathology laboratory (Dorevitch Pathology Labs, Australia) using standard clinical assays. Differences in biochemical markers from baseline will be compared between groups to evaluate changes resulting from the intervention. Upon completion of the OGTT, participants will be provided with the same standardised meal they received for pre-testing.

### Muscle strength assessment

#### Hand grip strength

Hand grip strength will be assessed with a digital handheld dynamometer (Jamar^®^ Plus+ Digital Hand Dynamometer, Sammons Preston, Bolingbrook, IL), to assess changes throughout the intervention and to assess sarcopenia risk [9]. Measurements will be taken with the participant standing, elbow positioned at their side and flexed at 90°, with the wrist in a neutral position. Participants will be instructed to squeeze the dynamometer with maximum effort using their dominant hand. This procedure will be repeated three times, with a one-minute rest between attempts. The highest recorded value from the dominant hand will be used for analysis [45].

#### 3-repetition maximum (3-RM) testing

Participants will perform a three-repetition maximum (3-RM) test to assess changes in both upper and lower body strength over the course of the intervention. Upper body strength will be evaluated using a fixed, pin-loaded chest press machine (Impulse^®^ IT9501 Chest Press, Impulse Fitness, Qingdao, China), while lower body strength will be assessed using a plate-loaded leg press machine (Body Iron^®^ Leg Press Machine, Body Iron, Melbourne, Australia). Prior to testing, participants will undergo a brief familiarisation with each resistance machine to ensure correct technique and safety. A standardised warm-up protocol will be completed, consisting of 8, 5, and 3 repetitions at 50%, 75%, and 95% of predicted 3-RM, respectively, to prepare for the 3-RM attempts [46, 47]. Participants will perform 3-RM attempts with a 2.50 kg (1.25 kg each side) minimum load increase each testing set, until 3-RM is reached. Participants will rest for 3–5 min between sets [48] and will be allowed to have 2 re-attempts at a failed lift. The outcome will be the maximal load (in kilograms) successfully lifted for three consecutive repetitions, recorded separately for chest press and leg press exercises [49, 50].

### Submaximal Incremental Bike Test (VO_2_peak)

To track changes in cardiorespiratory fitness participants will undergo a sub-maximal graded exercise test on a cycle ergometer (Lode Excaliber Sport, Lode Ergometry, Groningen, Netherlands). The test will begin at 10 watts (W), with increments of 10 watts (W) every minute until participants achieve a rate of perceived exertion (RPE) of 15 on the Borg scale, or are unable to maintain a cadence of ≥ 60 revolutions per minute (RPM) for more than 30 s. The test will also be terminated if participants request to stop or if any clinical contraindications to continued exercise arise. The highest workload completed at RPE 15 will be recorded as peak power output and used to estimate VO_2peak_. This submaximal RPE-based cycling protocol has demonstrated acceptable validity and safety in older adult populations [51–53].

### Blood pressure monitoring and safety procedures

Resting blood pressure (BP) will be assessed once participants are seated and have rested quietly for at least 5 min, prior to commencing the OGTT and exercise testing. BP will be measured using an automatic blood pressure monitor (Welch Allyn ProBP 3400 Series, NY, USA), in accordance with standard clinical guidelines [54]. If resting systolic BP is ≥ 160 mmHg, BP will be re-measured on two additional occasions (total of three measurements), with at least 1–2 min between measurements. If systolic BP remains consistently ≥ 160 mmHg across all readings, testing will be terminated and the participant will be advised to follow up with their general practitioner (GP) and obtain medical clearance prior to proceeding with the 10-week exercise intervention. If systolic BP falls below 160 mmHg in subsequent measurements, testing may proceed with caution.

Blood pressure will also be measured immediately before and immediately after the submaximal bike test (OMRON HEM-7120, OMRON Healthcare Co., Ltd, Kyoto, Japan). If systolic BP is ≥ 160 mmHg at rest prior to exercise, and remains elevated following two confirmatory measurements, the testing session will be terminated and the participant referred to their GP for further investigation.

Participants will also be monitored for hypotension, defined as BP ≤ 90/60 mmHg [54–56]. In such cases, testing will proceed with caution, and participants will be closely monitored for symptoms including dizziness, light-headedness, faintness, or loss of balance. If any of these symptoms are present, OGTT and exercise testing will not proceed, and participants will be advised to seek medical follow-up.

### Submaximal exercise protocol

#### Dietary intake monitoring

Participants will be asked to fill out a 3-day food diary using the app Easy Diet Diary (Xyris Pty Ltd, 2024) on their own personal phones for 2- weekdays and 1 weekend day. They will be asked to complete the food diary at baseline, week 2, 5, 8 and 12 (see Fig. 1). A two-page instructional guide will be provided, and a member of the research team will explain how to accurately record dietary intake using the app [57]. Dietary intake data will be analysed using *FoodWorks.online* software (Version 2.0 Professional; *Xyris Pty Ltd*, 2026). The total average daily energy, macronutrients (carbohydrates, protein and fats) and fibre intakes will be analysed. Group comparisons and within-participant changes will be evaluated to identify any significant dietary modifications over the intervention period.

### Appetite, hunger, and supplement acceptability

#### Appetite and hunger ratings

Participants will complete validated appetite questionnaires administered via REDcap. Appetite and hunger assessments will be conducted at multiple time points (see Fig. 1): in a fasted state, immediately post-training, post-recovery drink, and two hours post-recovery drink. This is to evaluate acute satiety effects of the supplement post-exercise. Appetite and hunger will be measured using a 100-mm visual analogue scale (VAS) with opposing anchors (“not at all” at 0 and “extremely” at 100). The questionnaire comprises nine items assessing subjective appetite sensations. Example items include: “How strong is your feeling of hunger?” and “How strong is your urge to eat?” (see Appendix 2 for full item list).

#### Supplement acceptability

Acceptability of the protein and placebo supplements will be assessed via a structured questionnaire administered through REDcap, completed at the same time points as the appetite visual analogue scale (VAS) assessments (i.e., fasted, post-training, post-recovery drink, and two hours post-recovery drink; see Fig. 1). The questionnaire includes a series of 7-point Likert scale items evaluating various dimensions of acceptability, including taste, ease of preparation, perceived effort, and overall satisfaction. For example, participants will rate statements such as “How much do you like the taste of the supplement?” and “How easy is it for you to prepare the supplement?” with anchors ranging from 1 (“not at all” or “extremely difficult”) to 7 (“extremely” or “extremely easy”), depending on the question context. In addition, participants will complete a 10-point scale to assess their likelihood of continued use of the supplement. Anchors range from 1 (“I would take this supplement every opportunity I had”) to 10 (“I have never tried this supplement”), enabling evaluation of habitual acceptability and intention to use. The full Supplement Acceptability questionnaire is provided in Appendix 3.

### Interventions

#### Experimental groups

The experimental groups will be provided with a defatted almond protein powder (Provided by Harris Woolf Almonds; California, United States of America) as part of their dietary intervention. They will be asked to consume this supplement once daily with their usual breakfast and additionally after each supervised training session (2 days per week). For the daily serving, participants will be instructed to mix a pre-packaged single-dose unit with 250 ml of provided almond milk and chocolate powder (the total drink providing: 1127.6 Kj, 18.3 g protein, 28.2 g carbohydrate, 7.0 g fat and 8.2 g fibre; see appendix 4). This shake will be consumed with breakfast, as breakfast is typically suboptimal for protein for this demographic [19]. On training days, participants will consume a larger post-exercise serving consisting of 56 g of defatted almond meal and 300 ml of almond milk and 30 g of chocolate powder, providing a total of 1724.4 kJ, 29.4 g protein, 43.1 g carbohydrates and 13.0 g fibre. Details of the supplement and placebo compositions are provided in Appendix 4.

Participants in the placebo group will be provided an energy-matched maltodextrin drink to consume for breakfast and post training also mixed with provided almond milk (See Appendix 4). Maltodextrin was selected as the placebo comparator as it can be easily energy-matched without including other confounding nutrients (e.g., protein, fat or fibre). Once prepared with almond milk, both the intervention and placebo drinks are similar in appearance, colour, and volume, presenting as a light, opaque beverage. While minor differences in texture or mouthfeel may be perceptible due to the differing nutrient composition, participants are not informed of these differences, and no information is provided that would enable identification of group allocation.

All supplements are provided in single dose black bags labelled either A or B; to help maintain blinding, participants will be instructed to prepare the shakes with a measured amount of almond milk themselves to ensure consistency in preparation and consumption. On supervised training days, participants will be provided with their post-exercise supplement immediately following the completion of their session. The supervising trainer will observe consumption to ensure the supplement is consumed in full prior to the participant leaving the facility. Participants will be asked to not discuss the supplement with other participants that may be in the same room.

Compliance will be monitored through paper-based supplement logs completed by the participants at home, together with the return of all the empty and unused sachets. Participants will be reminded on a weekly basis during their exercise sessions to consume their daily supplements. Exercise session attendance will also be monitored, and any missing sessions will be noted. At the end of the study period both supplement and exercise compliance will be reported.

Participants will be advised to immediately report any adverse events, new medical conditions, or symptoms that arise during the trial period. The allocated intervention or comparator will be discontinued or modified if:

1. The participant experiences an adverse reaction deemed related to the supplement (e.g., gastrointestinal distress, allergic reaction).
2. The participant is advised by their treating healthcare provider to stop supplementation for medical reasons.
3. The participant requests withdrawal from supplementation for any reason.

All adverse events and reasons for discontinuation will be documented, and participants who discontinue the supplement will then withdraw from the study. A Data Monitoring Committee will not be established for this trial. This is justified by the low-risk nature of the intervention, the short trial duration, and the limited number of participants. Instead, safety monitoring will be conducted internally by the study Principal investigator (DLH) and researcher (ZH). All adverse events (AEs) and any safety concerns will be reviewed on an ongoing basis. The PI will maintain oversight of trial safety and has final authority to terminate, pause, or modify the trial in response to safety issues, adverse event trends, or emerging ethical concerns.

### Resistance and high-intensity training session

The supervised training intervention will commence in Week 2 of the study and continue for the full intervention period (10-weeks in total). Participants will attend two supervised training sessions per week on non-consecutive days, with a minimum of 48 h between sessions to allow for recovery. Each participant will train at a consistent time of day throughout the study, selecting either a morning session (7:00–9:00 AM) or afternoon session (2:00–5:00 PM) based on availability and preference. All training sessions will be fully supervised by either an accredited exercise physiologist or a certified personal trainer to ensure correct technique, monitor safety, and provide standardised instruction across sessions.

The resistance training protocol employed in this study is informed by the American College of Sports Medicine (ACSM) position stand on progressive resistance training for older adults and is designed to elicit improvements in skeletal muscle mass, strength, and functional capacity [50]. Comparable protocols have consistently demonstrated efficacy in enhancing muscle hypertrophy, neuromuscular performance, and physical function in older populations [12, 50].

Each training session will comprise six exercises, with the specific movements alternating across two distinct sessions (Table 1). All exercises will be performed using a controlled 2-1-2 repetition tempo (2 s eccentric, 1-second pause, 2 s concentric) to increase time under tension and promote motor control. Standardised rest intervals of 60–90 s between sets, and 2–3 min will be allotted between exercises (Tables 1 and 2).

**Table 1:** Outline of resistance exercise protocol

Session 1	Session 2
1. Leg press	1. Leg press
2. Seated leg curl	2. Seated leg curl
3. Leg extensions	3. Leg extensions
4. Seated row	4. Lat pulldown
5. Machine chest press	5. Machine chest-press
6. Standing DB shoulder press	6. Seated DB shoulder press

Notes:

• Exercises performed at 2-1-2 tempo (ECC – ISO – CON)

• Rest 60–90 s between each set

• Rest 2–3 min between each exercise

**Table 2:** Outline of progression of sets, reps and intensity of resistance training protocol

	Sets	Reps	Intensity
Weeks 1–2	2	10–12	65–70% 1RM or 1–2 RIR
Weeks 3–4	3	10–12	70–75% 1RM or 1–2 RIR
Weeks 5–6	3	8–10	75–80% 1RM or 1–2 RIR
Weeks 7–8	4	8–10	75–80% 1RM or 1–2 RIR
Weeks 9–10	4	10–12	70–75% 1RM or 1–2 RIR

Training loads for the leg press and machine chest press will be prescribed based on each participant’s 3-RM, assessed during the pre-intervention testing [58]. For the remaining exercises, loads will be estimated and adjusted according to participant performance and feedback using estimated 1-RM and Repetitions in Reserve (RIR)-based progression. RIR reflects the number of repetitions a participant estimates they could have completed beyond the final repetition of a set, before reaching muscular failure. For example, a RIR of 1 means the participant believes they could have performed one additional reps with proper form. This method allows for a practical and individualised approach to adjusting training intensity, helping to ensure that each participant trains within a consistent and safe effort range [59]. Loads will be adjusted throughout the intervention to maintain the target RIR range and optimise training stimulus. Two weekly sessions of resistance training, consisting of multi-joint and full-body compound exercises at moderate intensity (e.g., 60–70% 1-RM), are sufficient to promote moderate yet significant increases in muscle mass in postmenopausal women [60].

#### HIIT protocol

The HIIT protocol was adapted from previous training studies in postmenopausal women that prescribed cycling intensity between 75 and 110% of PPO in overweight or obese women and ranged in duration for 3–12 weeks [61–63]. The HIIT protocol will be performed on a cycle ergometer which is outlined in Table 3. Each session begins with a 3-minute warm-up at 20 W, followed by 10 alternating bouts of high-intensity and recovery intervals. Each high-intensity interval consists of 60 s of cycling at 70–90% of the participant’s estimated peak power output, followed by 60 s of recovery. A 2-minute cool-down at 20 W concludes the session. Work intensity will progress every two weeks, beginning at 70% of PPO in Weeks 1–2 and increasing incrementally to 90% PPO by Weeks 9–10.

**Table 3:** HIIT protocol structure

Warm-up	3 min @ 20 W
HIIT intervals	10 × (60 s @ 70–90% PPO, 60 s active recovery)
Cool-down	2 min @ 20 W
Progression	70% PPO (Weeks 1–2) → 90% PPO (Weeks 9–10) *increasing by 5% each two weeks

#### Concomitant care

Participants will be instructed to maintain their habitual diet and physical activity patterns outside of the supervised training sessions for the duration of the study. Initiation of new structured exercise, high-intensity training or formal dietary interventions (e.g., weight loss programs, protein or fibre supplements) outside of the prescribed intervention will also be discouraged. Usual medical care and medications for unrelated health conditions will be permitted, provided they are stable for at least 3 months prior to enrolment and remain unchanged during the study period.

### Randomisation and blinding

#### Sequence generation

The random allocation sequence will be generated by an independent researcher not otherwise involved in participant enrolment, intervention delivery, or outcome assessment. A stratified block randomised approach will be used. Participants who meet inclusion criteria and consent to participate will be randomly allocated to either the experimental or placebo group after the first laboratory visit.

Randomisation will be stratified according to the following variables:

1. Hormone replacement therapy use (Yes/No)
2. Appendicular skeletal muscle mass Index (Low/High), determined by baseline DXA scan and classified according to the European Working Group on Sarcopenia in Older People (EWGSOP) cut-offs [9]

#### Allocation concealment and blinding

Allocation will be concealed using the independently generated randomisation schedule. Participants, intervention providers (exercise supervisors), outcome assessors, and data analysts will be blinded to group allocation throughout the study. The intervention (defatted almond protein powder) and placebo (maltodextrin) supplements will be identical in appearance, packaging, and flavour (chocolate).

Blinding will be maintained until data collection is complete and the primary analysis has been finalised. Emergency unblinding will only occur if required for participant safety, as determined by the Chief Investigator. For further blinding participants will be asked to refrain from discussing their supplements to the assessors or other participants during the study.

### Data collection, management, and analysis

#### Data collection and management

All data collected will be undertaken by trained researchers that are familiar with the study protocols. Training will include standardisation of measurement techniques, use of calibrated equipment, and administration of questionnaires to minimise inter-assessor variability. Where applicable, duplicate measurements will be taken (e.g., blood glucose) and the average recorded. Objective outcomes include DXA-derived body composition, OGTT-derived glucose and insulin responses, blood lipids, muscle strength (3-RM testing and hand grip dynamometry), and cardiorespiratory fitness (submaximal cycle test). Subjective outcomes include validated appetite visual analogue scales and supplement acceptability questionnaires administered via REDCap. Dietary intake will be assessed using 3-day food diaries recorded via the Easy Diet Diary app and analysed using FoodWorks software. All instruments and procedures are described in detail within the Methods section and relevant appendices.

#### Data monitoring

Research assistants that are blinded to the supplement allocation will be trained on how to collect data. Data for body composition, OGTT (blood glucose) and exercise testing will be collected at the same location during the study period. Data will be collected using de-identified, participant coded data collection sheets. These data will then be transferred electronically on password protected computers and the data sheets are stored in a locked filing cabinet.

While data is being collected, all data will be checked for omissions or errors by the project manager who will maintain a data diary of any issues that arise with the data. The data then will be checked again by the principal investigator (PI) and statistician prior to any analysis. The information collected from this study will be kept until the end of the project and then placed in archives for a minimum of 5 years from the final publication of the findings. Upon completion of the study all the data will be transferred to the Deakin Research Data Store, where all data will be de-identified and may be used for future studies.

The data will remain de-identified, with only the principal investigator retaining the coding file necessary for participant re-identification. Future access to the data will require permission from the PI, who will provide access to de-identified data files on the RDS. Any new research projects utilising this data will be submitted for ethical approval by the relevant university committee. Participants will be informed of the potential for their data to be used in future projects through the Plain Language Statement, with assurance that only de-identified data will be shared with other research team members.

#### Data analysis

Participant withdrawals and missing data will be documented, with primary analyses conducted under an intention-to-treat (ITT) framework and secondary per-protocol analyses based on predefined adherence criteria. A secondary, per-protocol analysis will be carried out to include only participants that attend ≥ 80% of their training sessions and consumed ≥ 80% of their supplement over the course of the study. Between-group comparisons will be conducted using linear models, adjusting for baseline values. Outcome variables will be log-transformed where necessary to meet the assumptions of parametric testing. Within-group changes will be evaluated using appropriate statistical tests, such as mixed-models. All statistical analyses will be two-tailed, with significance set at *p* < .05. Multiple imputation by chained equations will be used to handle missing data.

#### Sample size and statistical power

As no established minimal clinically important difference exists for fat-free mass (FFM) or lean mass, as an alternative, sample size was justified on several factors: (1) based on biological aging trajectories; older adults typically experience a decline of approximately 0.5–1.0 kg per year in FFM [64, 65], equating to an estimated 1–2% annual loss. A 2.0% increase in lean mass in the intervention group may therefore be interpreted as a reversal of two years’ worth of age-related muscle loss; (2) in similar studies in this age group, the non-supplemented group may see a non-significant improvement of their lean mass of ~ 0.2–0.4% following 12-24-weeks of resistance training [66], whilst the intervention group supplemented with protein may improve lean mass by a magnitude of 2.4% [67]. Similarly, our own data with strength training 2x week in women supplemented with protein demonstrated an increase in lean mass of 2.7% [44]. The study was therefore powered to detect a between-group difference of 2.0% in FFM change (control: 0.4%; supplemented: 2.4%). Assuming a standard deviation of 2.2% (calculated from raw data from Huschtscha, Silver [44], this requires a sample size of 42 participants (21 per group) to achieve 80% power at a at α = 0.05 and accounting for an anticipated 10% dropout rate we will aim to recruit 48 participants in total.

## Discussion

A key strength of this study protocol is the use of a rigorously designed, placebo-controlled, double-blinded randomised controlled trial to evaluate the effects of defatted almond protein powder supplementation in combination with resistance and HIIT on musculoskeletal mass and function, and markers of cardiometabolic health in postmenopausal women. This study addresses a timely and clinically relevant question given the growing interest in plant-based sources of protein, recommendations to reduce animal protein intake, the expanding ageing population and the unique health challenges experienced by postmenopausal women [68].

The exercise intervention is designed to target improvements in muscular strength, cardiovascular fitness, and body composition, supported by prior research in similar populations [69]. APP supplementation, consisting of a daily shake at breakfast and a post-exercise shake on training days (2x days/week), provides a meaningful increase in both daily protein and fibre intakes (Appendix 4). On average, this equates to an increase of approximately 26 g/day of protein and 12 g/day of fibre across the week. National survey data, indicate that the average intake of protein for Australians females aged 19–50 years is 79 g/day [19]. With the APP supplementation, the additional protein provided (~ 26 g/day) would raise the average total protein intake for a postmenopausal woman to ~ 105 g/day. This aligns with the recommended intake of 1.2–1.6 g/kg/day for older adults and exceeds the current average intake of 1.1 g/kg/day, assuming an average body mass of ~ 71 kg [19].

Additionally, the intervention increases fibre intake by 12 g/day, from an average of ~ 21 g/day to 33 g/day. Thus, most participants should exceed the AI of 25 g/day and approach or exceed the 28 g/day target [27]. By comparison, the placebo condition provides only 2.4 g of fibre, mainly coming from the chocolate powder and almond milk, reinforcing the markedly greater fibre benefit of APP. Dietary fibre, particularly from nuts and other plant foods, is associated with reduced risk of cardiovascular disease, improved lipid profiles, better glycaemic control, and enhanced gut health [31–33]. Further evidence supports the cardiometabolic benefits of almond consumption in postmenopausal women, particularly on improvements in blood lipid profiles when replacing carbohydrate snacks with 30–73 g of almonds daily [70–72]. The average increased APP protein dose is equivalent to ~ 85–100 g of whole almonds but due to the defatting may offer the benefits of almonds within a smaller “calorie budget.”

The use of plant-based protein powder, particularly one derived from almonds, provides a novel aspect of this study. While soy [73], whey [34, 74, 75], and pea protein [76] isolates have been more widely studied, APP provides a sustainable, nutrient-dense alternative. Unlike many other protein supplements, which are usually refined concentrates or isolates, APP retains dietary fibre and other nutrients (e.g., calcium, iron and monounsaturated fatty acids) potentially enhancing the protein matrix effect [77, 78]. This characteristic may be particularly relevant for older women, whose habitual diets often fail to meet optimal intakes for both protein and fibre [19, 79].

Another important strength of this study is the use of an energy-matched, but nutrient- distinct placebo comparator, which allows the effects of protein–fibre–micronutrient matrix of defatted almond protein powder to be isolated from total energy intake.

Despite these strengths, one limitation is the lack of full control over the participants’ diet and physical activity outside the intervention. Although participants will be advised to maintain their usual dietary habits and physical activity outside of the intervention sessions, it is not feasible to fully standardise or control these variables over the 10-week period. In addition, because of the nutrient-distinct design, the APP supplement differs substantially in nutritional composition from the placebo, and therefore greater improvements in the APP group are expected given the nutritional differences between supplements. We expect that the APP protein group may experience a reduction in appetite, potentially leading to an overall decrease in energy intake and unintentional weight loss, which is what is hypothesised considering the unique nutrient profile. Additionally, by including the food diaries at various time periods during the study we hope to capture any changes in intakes that may result from changes in appetite and report accordingly.

Additionally, the use of maltodextrin was selected because it allows the control beverage to be energy-matched to the APP supplement without providing protein, fibre, or micronutrients that could confound the comparison. An energy-matched placebo is optimal in this context to ensure that any between-group differences can be more confidently attributed to the protein–fibre–micronutrient matrix of APP rather than differences in total energy intake.

This study is designed to explore the combined effects of a defatted almond protein powder supplementation and structured exercise on key indicators of musculoskeletal and cardiometabolic health in postmenopausal women. By providing a novel, plant-based protein source that also delivers meaningful amounts of dietary fibre, the intervention addresses two common nutritional gaps in this population, insufficient protein to support muscle maintenance and inadequate fibre for cardiometabolic health. When paired with evidence-based training modalities, this combination has the potential to improve muscle mass, strength, and function while supporting healthy lipid profiles, glycaemic control, and gut health. Despite the limitations outlined, the findings from this study may help inform dietary and exercise guidelines, contributing to the development of targeted interventions that support healthy ageing in women.

## Electronic Supplementary Material

Supplementary Material 1.

## Data Availability

The datasets used and/or analysed during the current study are available from the corresponding author on reasonable request. The research team plans to disseminate the findings of this clinical trial at appropriate conferences, and the results will be published via a peer-reviewed journal.

## Abbreviations

ABC: Almond Board of California
APP: Almond Protein Powder
ASM: Appendicular skeletal muscle mass
BM: Body mass
BMI: Body mass index
CV: Coefficient of variation
DB: Dumbbell
DXA: Dual-energy X-ray absorptiometry
EAR: Estimated Average Requirement
EWGSOP: European Working Group on Sarcopenia in Older People
FFM: Fat-free mass
HDL-C: High-density lipoprotein cholesterol
HIIT: High-intensity interval training
HOMA-IR: Homeostatic Model Assessment of Insulin Resistance
HRT: Hormone replacement therapy
IPAN: Institute for Physical Activity and Nutrition
ITT: Intention-to-treat
LDL-C: Low-density lipoprotein cholesterol
MPS: Muscle protein synthesis
PAR-Q: Physical Activity Readiness Questionnaire
PPO: Peak power output
PLS: Plain language statement
OGTT: Oral glucose tolerance test
REDcap: Research Electronic Data Capture
RIR: Repetitions in reserve
RMR: Resting metabolic rate
RPE: Rate of perceived exertion
RPM: Revolutions per minute
SCFA: Short-chain fatty acids
SPIRIT: Standard Protocol Items: Recommendations for Interventional Trials
TC: Total cholesterol
TG: Triglycerides
USG: Urine specific gravity
VAS: Visual analogue scale
VO2peak: Peak oxygen uptake
W: Watts
1-RM/3-RM: One/Three repetition maximum

## References

[1] WHO, Ageing. and health 2022. Available from: https://www.who.int/news-room/fact-sheets/detail/ageing-and-health.

[2] Hosseinpoor AR, Stewart Williams J, Jann B, Kowal P, Officer A, Posarac A, et al. Social determinants of sex differences in disability among older adults: a multi-country decomposition analysis using the World Health Survey. Int J Equity Health. 2012;11:1–8. 22958712 10.1186/1475-9276-11-52 PMC3463479

[3] Patwardhan V, Gil GF, Arrieta A, Cagney J, DeGraw E, Herbert ME, et al. Differences across the lifespan between females and males in the top 20 causes of disease burden globally: a systematic analysis of the Global Burden of Disease Study 2021. Lancet Public Health. 2024;9(5):e282–94. 38702093 10.1016/S2468-2667(24)00053-7 PMC11080072

[4] Fenton A, Weight. Shape, and Body Composition Changes at Menopause. J Mid-life Health. 2021;12(3):187–92. 10.4103/jmh.jmh_123_21 PMC8569454 34759699

[5] Kodoth V, Scaccia S, Aggarwal B. Adverse Changes in Body Composition During the Menopausal Transition and Relation to Cardiovascular Risk: A Contemporary Review. Womens Health Rep (New Rochelle). 2022;3(1):573–81. 35814604 10.1089/whr.2021.0119 PMC9258798

[6] Huang S, Gongye R, Zou S, Hee JY, Tang K. Menopausal status, age at menopause and risk of all-cause mortality among Chinese women: findings from a 10-year prospective study. BMJ Public Health. 2023;1(1). 10.1136/bmjph-2023-000332. 10.1136/bmjph-2023-000332 PMC11816857 40017861

[7] Abdulnour J, Doucet E, Brochu M, Lavoie J-M, Strychar I, Rabasa-Lhoret R, et al. The effect of the menopausal transition on body composition and cardiometabolic risk factors: a Montreal-Ottawa New Emerging Team group study. Menopause. 2012;19(7):760–7. 22395454 10.1097/gme.0b013e318240f6f3

[8] Després JP. Body fat distribution and risk of cardiovascular disease: an update. Circulation. 2012;126(10):1301–13. 22949540 10.1161/CIRCULATIONAHA.111.067264

[9] Cruz-Jentoft AJ, Bahat G, Bauer J, Boirie Y, Bruyère O, Cederholm T, et al. Sarcopenia: revised European consensus on definition and diagnosis. Age Ageing. 2019;48(1):16–31. 30312372 10.1093/ageing/afy169 PMC6322506

[10] Hulteen RM, Marlatt KL, Allerton TD, Lovre D. Detrimental Changes in Health during Menopause: The Role of Physical Activity. Int J Sports Med. 2023;44(6):389–96. 36807278 10.1055/a-2003-9406 PMC10467628

[11] Boutot ME, Purdue-Smithe A, Whitcomb BW, Szegda KL, Manson JE, Hankinson SE, et al. Dietary protein intake and early menopause in the Nurses’ Health Study II. Am J Epidemiol. 2018;187(2):270–7. 28992246 10.1093/aje/kwx256 PMC5860152

[12] Huschtscha Z, Parr A, Porter J, Costa RJ. The effects of a high-protein dairy milk beverage with or without progressive resistance training on fat-free mass, skeletal muscle strength and power, and functional performance in healthy active older adults: a 12-week randomized controlled trial. Front Nutr. 2021;8:644865. 33816540 10.3389/fnut.2021.644865 PMC8010144

[13] Sá KMM, da Silva GR, Martins UK, Colovati MES, Crizol GR, Riera R, et al. Resistance training for postmenopausal women: systematic review and meta-analysis. Menopause. 2023;30(1):108–16. 36283059 10.1097/GME.0000000000002079

[14] Asikainen TM, Kukkonen-Harjula K, Miilunpalo S. Exercise for health for early postmenopausal women: a systematic review of randomised controlled trials. Sports Med. 2004;34(11):753–78. 15456348 10.2165/00007256-200434110-00004

[15] da Silva JLJ, Orsatti FL, Margato LR, Silva RM, de Sousa WG, de Oliveira Assumpção C, et al. Optimizing resistance training for body recomposition in postmenopausal women. Sport Sci Health. 2024;20(3):983–94.

[16] Nunes PRP, Barcelos LC, Oliveira AA, Furlanetto Junior R, Martins FM, Orsatti CL, et al. Effect of resistance training on muscular strength and indicators of abdominal adiposity, metabolic risk, and inflammation in postmenopausal women: controlled and randomized clinical trial of efficacy of training volume. Age. 2016;38:1–13. 26984105 10.1007/s11357-016-9901-6 PMC5005909

[17] Mandrup CM, Egelund J, Nyberg M, Lundberg Slingsby MH, Andersen CB, Løgstrup S, et al. Effects of high-intensity training on cardiovascular risk factors in premenopausal and postmenopausal women. Am J Obstet Gynecol. 2017;216(4):e3841–11. 10.1016/j.ajog.2016.12.017 28024987

[18] Yarış S, Yetgin MK, Agopyan A, Kasımay Ö, Gedikbaşı A, Demirci H, et al. Impact of High-Intensity Interval Training on Bone Metabolism and the Metabolic and Hormonal Profiles of Postmenopausal Women. Istanbul Med J. 2025;26(1):5–10. 10.4274/imj.galenos.2024.08505.

[19] Noakes M. Protein balance: New concepts for protein in weight management. Australia: CSIRO; 2018.

[20] Silva TR, Spritzer PM. Skeletal muscle mass is associated with higher dietary protein intake and lower body fat in postmenopausal women: a cross-sectional study. Menopause. 2017;24(5):502–9. 27922938 10.1097/GME.0000000000000793

[21] Australian Health Survey: Nutrition First Results – Foods and Nutrients, 2011-12. 2015. Available from: https://www.abs.gov.au/ausstats/abs@.nsf/lookup/4338.0main+features212011-13. Cited 8/07/2025.

[22] Morton RW, Traylor DA, Weijs PJ, Phillips SM. Defining anabolic resistance: implications for delivery of clinical care nutrition. Curr Opin Crit Care. 2018;24(2):124–30. 29389741 10.1097/MCC.0000000000000488

[23] Phillips SM. Current concepts and unresolved questions in dietary protein requirements and supplements in adults. Front Nutr. 2017;4:13. 28534027 10.3389/fnut.2017.00013 PMC5420553

[24] Huschtscha Z, Porter J, Parr A, Costa RJ. Protein amount, quality and distribution in active older adults and its effects on outcomes of fat free mass, skeletal muscle strength and power. Int J Sports Sci. 2021;11(1):6–17. 10.5923/j.sports.20211101.02.

[25] Huschtscha Z, Parr A, Porter J, Costa RJS. Sarcopenic Characteristics of Active Older Adults: a Cross-Sectional Exploration. Sports Med - Open. 2021;7(1):32. 33999277 10.1186/s40798-021-00323-9 PMC8128944

[26] Fayet-Moore F, Cassettari T, Tuck K, McConnell A, Petocz P. Dietary fibre intake in Australia. Paper I: associations with demographic, socio-economic, and anthropometric factors. Nutrients. 2018;10(5):599. 29751656 10.3390/nu10050599 PMC5986479

[27] Menu A. Australian Health Survey: Nutrition First Results—Foods and Nutrients. Canberra, Australia: Australian Bureau of Statistics; 2011.

[28] Tsitsou S, Athanasaki C, Dimitriadis G, Papakonstantinou E. Acute effects of dietary fiber in starchy foods on glycemic and insulinemic responses: a systematic review of randomized controlled crossover trials. Nutrients. 2023;15(10):2383. 37242267 10.3390/nu15102383 PMC10223420

[29] Slavin J. Health aspects of dietary fibre. Fibre-Rich Wholegrain Foods Improv Qual. 2013;237:61–75.

[30] Simpson HL, Campbell BJ. dietary fibre–microbiota interactions. Aliment Pharmacol Ther. 2015;42(2):158–79. 26011307 10.1111/apt.13248 PMC4949558

[31] Bulsiewicz WJ. The importance of dietary fiber for metabolic health. Am J Lifestyle Med. 2023;17(5):639–48. 37711348 10.1177/15598276231167778 PMC10498976

[32] Reynolds AN, Akerman A, Kumar S, Diep Pham HT, Coffey S, Mann J. Dietary fibre in hypertension and cardiovascular disease management: systematic review and meta-analyses. BMC Med. 2022;20(1):139. 35449060 10.1186/s12916-022-02328-x PMC9027105

[33] Threapleton DE, Greenwood DC, Evans CE, Cleghorn CL, Nykjaer C, Woodhead C, et al. Dietary fibre intake and risk of cardiovascular disease: systematic review and meta-analysis. BMJ. 2013;347:f6879. 10.1136/bmj.f6879. 10.1136/bmj.f6879 PMC3898422 24355537

[34] Li C, Meng H, Wu S, Fang A, Liao G, Tan X, et al. Daily supplementation with whey, soy, or whey-soy blended protein for 6 months maintained lean muscle mass and physical performance in older adults with low lean mass. J Acad Nutr Dietetics. 2021;121(6):1035–48. e6. 10.1016/j.jand.2021.01.006 33612439

[35] Phillips SM, Tang JE, Moore DR. The role of milk-and soy-based protein in support of muscle protein synthesis and muscle protein accretion in young and elderly persons. J Am Coll Nutr. 2009;28(4):343–54. 20368372 10.1080/07315724.2009.10718096

[36] Fraser GE, Bennett HW, Jaceldo KB, Sabaté J. Effect on body weight of a free 76 kilojoule (320 calorie) daily supplement of almonds for six months. J Am Coll Nutr. 2002;21(3):275–83. 12074256 10.1080/07315724.2002.10719221

[37] Maykish A, Nishisaka MM, Talbott CK, Reaves SK, Kristo AS, Sikalidis AK. Comparison of whey versus almond protein powder on nitrogen balance in female college students; the California almond protein powder project (CAlmond-P3). Int J Environ Res Public Health. 2021;18(22):11939. 34831691 10.3390/ijerph182211939 PMC8620843

[38] Lamb DA, Moore JH, Smith MA, Vann CG, Osburn SC, Ruple BA, et al. The effects of resistance training with or without peanut protein supplementation on skeletal muscle and strength adaptations in older individuals. J Int Soc Sports Nutr. 2020;17:1–17. 33317565 10.1186/s12970-020-00397-y PMC7734909

[39] Lacivita V, Derossi A, Caporizzi R, Lamacchia C, Speranza B, Guerrieri A, et al. Discover hidden value of almond by-products: Nutritional, sensory, technological and microbiological aspects. Future Foods. 2024;10:100398.

[40] Chan A-W, Boutron I, Hopewell S, Moher D, Schulz KF, Collins GS, et al. SPIRIT 2025 statement: updated guideline for protocols of randomised trials. BMJ. 2025;389:e081477. 40294953 10.1136/bmj-2024-081477 PMC12035670

[41] Organization WH. Physical status: The use of and interpretation of anthropometry. Report of a WHO Expert Committee: World Health Organization; 1995. 8594834

[42] Klein KR, Walker CP, McFerren AL, Huffman H, Frohlich F, Buse JB. Carbohydrate Intake Prior to Oral Glucose Tolerance Testing. J Endocr Soc. 2021;5(5):bvab049. 33928207 10.1210/jendso/bvab049 PMC8059359

[43] Nana A, Slater GJ, Stewart AD, Burke LM. Methodology review: using dual-energy X-ray absorptiometry (DXA) for the assessment of body composition in athletes and active people. Int J Sport Nutr Exerc Metab. 2015;25(2):198–215. 25029265 10.1123/ijsnem.2013-0228

[44] Huschtscha Z, Silver J, Gerhardy M, Urwin CS, Kenney N, Le VH, et al. The Effect of Palmitoylethanolamide (PEA) on Skeletal Muscle Hypertrophy, Strength, and Power in Response to Resistance Training in Healthy Active Adults: A Double-Blind Randomized Control Trial. Sports Medicine-Open. 2024;10(1):66. 38844675 10.1186/s40798-024-00732-6 PMC11156829

[45] Mehmet H, Yang AW, Robinson SR. Measurement of hand grip strength in the elderly: A scoping review with recommendations. J Bodyw Mov Ther. 2020;24(1):235–43. 31987550 10.1016/j.jbmt.2019.05.029

[46] NSCA-National Strength & Conditioning Association, editor. Essentials of strength training and conditioning. Human kinetics; 2021. p. 440.

[47] Ratamess NA, Alvar BA, Evetoch TK, Housh TJ, Kibler WB, Kraemer WJ, et al. Progression models in resistance training for healthy adults. Med Sci Sports Exercise: Official J Am Coll Sports Med. 2009;41(3):687–708. 10.1249/MSS.0b013e3181915670 19204579

[48] Kraemer WJ, Fleck SJ, Deschenes MR. Exercise physiology: Integrating theory and application: Second edition; 2014:p 1–525.

[49] McCurdy K, Langford GA, Cline AL, Doscher M, Hoff R. The reliability of 1-and 3RM tests of unilateral strength in trained and untrained men and women. J sports Sci Med. 2004;3(3):190. 24482597 PMC3905302

[50] Fragala MS, Cadore EL, Dorgo S, Izquierdo M, Kraemer WJ, Peterson MD, et al. Resistance training for older adults: position statement from the National strength and conditioning association. J Strength Conditioning Res. 2019;33(8). 10.1519/JSC.0000000000003230. 10.1519/JSC.0000000000003230 31343601

[51] Coquart JB, Garcin M, Parfitt G, Tourny-Chollet C, Eston RG. Prediction of maximal or peak oxygen uptake from ratings of perceived exertion. Sports Med. 2014;44(5):563–78. 24519666 10.1007/s40279-013-0139-5

[52] Zinoubi B, Zbidi S, Vandewalle H, Chamari K, Driss T. Relationships between rating of perceived exertion, heart rate and blood lactate during continuous and alternated-intensity cycling exercises. Biol Sport. 2018;35(1):29–37. 30237659 10.5114/biolsport.2018.70749 PMC6135975

[53] Hall M, Lima YL, Huschtscha Z, Dobson F, Costa RJ. What is real change in submaximal cardiorespiratory fitness in older adults? Retrospective analysis of a clinical trial. Sports Medicine-Open. 2022;8(1):59. 35482259 10.1186/s40798-022-00447-6 PMC9051008

[54] Selig SE, Levinger I, Williams AD, Smart N, Holland DJ, Maiorana A, et al. Exercise & Sports Science Australia Position Statement on exercise training and chronic heart failure. J Sci Med Sport. 2010;13(3):288–94. 20227917 10.1016/j.jsams.2010.01.004

[55] Freeman R, Wieling W, Axelrod FB, Benditt DG, Benarroch E, Biaggioni I, et al. Consensus statement on the definition of orthostatic hypotension, neurally mediated syncope and the postural tachycardia syndrome. Clin Auton Res. 2011;21(2):69–72. 21431947 10.1007/s10286-011-0119-5

[56] Severin R, Sabbahi A, Albarrati A, Phillips SA, Arena S. Blood Pressure Screening by Outpatient Physical Therapists: A Call to Action and Clinical Recommendations. Phys Ther. 2020;100(6):1008–19. 32232372 10.1093/ptj/pzaa034 PMC7462048

[57] Ltd XSAP. Quick guide to getting started with Easy Diet Diary: Easy Diet Diary Support; n.d. Available from: https://support.easydietdiary.com/hc/en-us/articles/360038828051-Quick-guide-to-getting-started-with-Easy-Diet-Diary-PDF.

[58] Brzycki M. Strength testing—predicting a one-rep max from reps-to-fatigue. J Phys Educ recreation dance. 1993;64(1):88–90.

[59] Helms ER, Cronin J, Storey A, Zourdos MC. Application of the repetitions in reserve-based rating of perceived exertion scale for resistance training. Strength Conditioning J. 2016;38(4):42–9. 10.1519/SSC.0000000000000218 PMC4961270 27531969

[60] Thomas E, Gentile A, Lakicevic N, Moro T, Bellafiore M, Paoli A, et al. The effect of resistance training programs on lean body mass in postmenopausal and elderly women: a meta-analysis of observational studies. Aging Clin Exp Res. 2021:1–12. 10.1007/s40520-021-01853-8 PMC8595144 33880736

[61] Clark A, De La Rosa AB, DeRevere JL, Astorino TA. Effects of various interval training regimes on changes in maximal oxygen uptake, body composition, and muscular strength in sedentary women with obesity. Eur J Appl Physiol. 2019;119(4):879–88. 30643959 10.1007/s00421-019-04077-x

[62] Smith-Ryan AE, Trexler ET, Wingfield HL, Blue MN. Effects of high-intensity interval training on cardiometabolic risk factors in overweight/obese women. J Sports Sci. 2016;34(21):2038–46. 26934687 10.1080/02640414.2016.1149609 PMC5010533

[63] Peishun C, Yu M, Yanjun L, Hua G, Hongli G, Wanrong Z. Effects of high-intensity interval exercise training on knee health and articular cartilage volume in patients with obesity: A comparative study between bicycle and treadmill groups. J Bodyw Mov Ther. 2024;40:472–5. 39593628 10.1016/j.jbmt.2024.04.053

[64] Goodpaster BH, Park SW, Harris TB, Kritchevsky SB, Nevitt M, Schwartz AV, et al. The Loss of Skeletal Muscle Strength, Mass, and Quality in Older Adults: The Health, Aging and Body Composition Study. Journals Gerontology: Ser A. 2006;61(10):1059–64. 10.1093/gerona/61.10.1059 17077199

[65] Hughes VA, Frontera WR, Roubenoff R, Evans WJ, Singh MAF. Longitudinal changes in body composition in older men and women: role of body weight change and physical activity1234. Am J Clin Nutr. 2002;76(2):473–81. 12145025 10.1093/ajcn/76.2.473

[66] Holm L, Olesen JL, Matsumoto K, Doi T, Mizuno M, Alsted TJ, et al. Protein-containing nutrient supplementation following strength training enhances the effect on muscle mass, strength, and bone formation in postmenopausal women. J Appl Physiol. 2008;105(1):274–81. 18467544 10.1152/japplphysiol.00935.2007

[67] Tieland M, van de Rest O, Dirks ML, van der Zwaluw N, Mensink M, van Loon LJ, et al. Protein supplementation improves physical performance in frail elderly people: a randomized, double-blind, placebo-controlled trial. J Am Med Dir Assoc. 2012;13(8):720–6. 22889730 10.1016/j.jamda.2012.07.005

[68] Willett W, Rockström J, Loken B, Springmann M, Lang T, Vermeulen S, et al. Food in the Anthropocene: the EAT–Lancet Commission on healthy diets from sustainable food systems. Lancet. 2019;393(10170):447–92. 30660336 10.1016/S0140-6736(18)31788-4

[69] González-Gálvez N, Moreno-Torres J, Vaquero-Cristóbal R. Resistance training effects on healthy postmenopausal women: a systematic review with meta-analysis. Climacteric. 2024;27(3):296–304. 38353251 10.1080/13697137.2024.2310521

[70] Richmond K, Williams S, Mann J, Brown R, Chisholm A. Markers of cardiovascular risk in postmenopausal women with type 2 diabetes are improved by the daily consumption of almonds or sunflower kernels: a feeding study. Int Sch Res Notices. 2013;2013(1):626414. 10.5402/2013/626414 PMC4045277 24959542

[71] Berryman CE, West SG, Fleming JA, Bordi PL, Kris-Etherton PM. Effects of daily almond consumption on cardiometabolic risk and abdominal adiposity in healthy adults with elevated LDL‐cholesterol: a randomized controlled trial. J Am Heart Association. 2015;4(1):e000993. 10.1161/JAHA.114.000993 PMC4330049 25559009

[72] Jenkins DJ, Kendall CW, Marchie A, Parker TL, Connelly PW, Qian W, et al. Dose response of almonds on coronary heart disease risk factors: blood lipids, oxidized low-density lipoproteins, lipoprotein (a), homocysteine, and pulmonary nitric oxide: a randomized, controlled, crossover trial. Circulation. 2002;106(11):1327–32. 12221048 10.1161/01.cir.0000028421.91733.20

[73] DeNysschen CA, Burton HW, Horvath PJ, Leddy JJ, Browne RW. Resistance training with soy vs whey protein supplements in hyperlipidemic males. J Int Soc Sports Nutr. 2009;6(1):8. 19284589 10.1186/1550-2783-6-8 PMC2660282

[74] Moon JM, Ratliff KM, Blumkaitis JC, Harty PS, Zabriskie HA, Stecker RA, et al. Effects of daily 24-gram doses of rice or whey protein on resistance training adaptations in trained males. J Int Soc Sports Nutr. 2020;17(1):60. 10.1186/s12970-020-00394-1. 33261645 10.1186/s12970-020-00394-1 PMC7706190

[75] Tang JE, Moore DR, Kujbida GW, Tarnopolsky MA, Phillips SM. Ingestion of whey hydrolysate, casein, or soy protein isolate: effects on mixed muscle protein synthesis at rest and following resistance exercise in young men. J Appl Physiol. 2009;107(3):987–92. 10.1152/japplphysiol.00076.200. 19589961 10.1152/japplphysiol.00076.2009

[76] Joy JM, Lowery RP, Wilson JM, Purpura M, De Souza EO, Wilson SM, et al. The effects of 8 weeks of whey or rice protein supplementation on body composition and exercise performance. Nutr J. 2013;12:1–7. 23782948 10.1186/1475-2891-12-86 PMC3698202

[77] Burd NA, Beals JW, Martinez IG, Salvador AF, Skinner SK. Food-First Approach to Enhance the Regulation of Post-exercise Skeletal Muscle Protein Synthesis and Remodeling. Sports Med. 2019;49(Suppl 1):59–68. 30671904 10.1007/s40279-018-1009-y PMC6445816

[78] Burd NA, Gorissen SH, van Vliet S, Snijders T, van Loon LJ. Differences in postprandial protein handling after beef compared with milk ingestion during postexercise recovery: a randomized controlled trial. Am J Clin Nutr. 2015;102(4):828–36. 26354539 10.3945/ajcn.114.103184

[79] Noll P, Campos C, Leone C, Zangirolami-Raimundo J, Noll M, Baracat E, et al. Dietary intake and menopausal symptoms in postmenopausal women: a systematic review. Climacteric. 2021;24(2):128–38. 33112163 10.1080/13697137.2020.1828854

